# Parallel/Opposed: IMRT QA using treatment log files is superior to conventional measurement‐based method

**DOI:** 10.1120/jacmp.v16i1.5385

**Published:** 2015-01-08

**Authors:** Nathan Childress, Quan Chen, Yi Rong

**Affiliations:** ^1^ Mobius Medical Systems Houston TX; ^2^ Department of Radiation Oncology University of Virginia Charlottesville VA; ^3^ Department of Radiation Oncology Ohio State University Wexner Medical Center Columbus OH USA

## I. INTRODUCTION

In the recent years, more and more researchers believe measurement‐based quality assurance (QA) method for intensity‐modulated radiotherapy (IMRT) is insensitive in detecting various types of failures.^1^, ^2^, ^3^ Machine delivery log‐file analysis has been proposed to be a more effective and efficient approach in verifying IMRT delivery accuracy in terms of gantry, collimator, jaws, and MLCs.^1^, ^4^, ^5^, ^6^, ^7^, ^8^, ^9^ However, whether log file measurements can replace conventional QA methods remains a major debate in current medical physics society. This is addressed as our first parallel/opposed topic.

Nathan Childress is parallel to the argument. Dr. Childress received his Ph.D. in Medical Physics in 2004 from University of Texas‐M.D. Anderson Cancer Center, his M.S. in Nuclear Engineering, and his B.S. in Chemical Engineering from the University of Missouri‐Columbia, both in 2001. His dissertation focused on bulk IMRT QA analysis and film dosimetry. He worked as a clinical physicist for six years at The Methodist Hospital before founding Mobius Medical Systems, LP in 2010. Mobius has designed and developed software packages that perform linear accelerator QA, treatment plan QA, and IMRT QA. He created and maintains www.medphysfiles.com, a site for the free sharing of files related to clinical medical physics. Dr. Childress is a Section Editor for the *JACMP* and is certified by the American Board of Radiology in Therapeutic Radiological Physics.

Quan Chen is opposing the argument. Dr. Chen obtained his Ph.D in medical physics from University of Wisconsin‐Madison, Madison, WI in 2004. He joined TomoTherapy Inc. the same year as a medical physicist, focusing on research and innovations. He joined University of Virginia in 2011 as an assistant professor in the radiation oncology department. His main research interests include tomotherapy, high‐performance computing, optimization and dose calculation, Monte Carlo, and innovative QA methods. He has authored and co‐authored over 50 papers in peer‐reviewed journals and holds seven granted patents.

### Dr. Nathan Childress (Mobius Medical Systems, LP)

The goal of IMRT QA is to ensure each patient receives safe and effective treatment. Linear accelerator log files, when used intelligently, are the optimal means of performing IMRT QA. Not only can log file‐based IMRT QA verify information transfer integrity and delivery performance, it can do so more accurately and efficiently than conventional methods.

Conventional IMRT QA methods of measuring the dose distribution in a plastic phantom are laborious, insensitive to some error types, and devoid of specificity. This leads some physicists to view IMRT QA as a tool to detect large machine calibration errors, whereas the true goal of IMRT QA is detecting errors specific to an individual patient's plan.

Conventional IMRT QA produces a single, integrated result with limited pathway to identify and remedy error sources. Instead of fixing root causes, physicists end up either aimlessly repeating IMRT QA measurements until they pass, or they spend copious amounts of time investigating individual components. The lack of sophisticated methods may be one reason why approximately 20% of institutions fail RPC heterogeneous phantom irradiations at 7%/4 mm criteria^(10)^ despite having acceptable conventional IMRT QA outcomes.

Performing IMRT QA using log files offers several advantages. First, the delivered 3D dose can be calculated from log files in the patient CT and compared directly with the prescribed treatment plan. Not only does this comparison validate information transfer from the planning system, but it also allows for a comprehensive and quantitative assessment of the impact of delivery performance on the three‐dimensional dose in a patient. Second, log files are produced each time the plan is delivered, meaning the method can be utilized during patient deliveries, in addition to a single pretreatment measurement. This extends the current IMRT QA paradigm to include the entire course of delivery. Third, the entire process can be completely automated, yielding a detailed analysis of dose in patient's anatomy available within minutes of delivery. This reduces the workload required for IMRT QA on the medical physics team, and enables them to more efficiently focus their effort on evaluating the QA outcome rather than producing it. Finally, a system that employs an accurate, independent dose calculation algorithm to recalculate the dose in the patient CT using both the planned values and the data contained in the log files allows for a clear separation of errors (treatment plan versus treatment delivery), thus allowing physicists to fix root causes of problems.

It is important to note that log file‐based IMRT QA must be augmented with independent commissioning measurements and a robust routine QA program (such as AAPM TG 142) to verify machine calibration. Once external systems are used to validate machine calibrations, the high temporal and spatial resolution of log files can identify patient‐specific errors and their sources almost automatically. Detection of certain errors in IMRT QA may then trigger additional machine‐specific tests.

The use of log files for IMRT QA, when intelligently utilized as part of a robust QA program, can give physicists more time and information to analyze the clinical impact of detected errors and effectively mitigate them.

### Dr. Quan Chen (Radiation Oncology, University of Virginia, Charlottesville, VA)

QA for IMRT using treatment log files has been developed recently on various treatment machines^5^, ^11^, ^12^ to address issues with conventional QA methods. Different from conventional QA methods, the log file‐based approach relies on the detailed knowledge of the actual treatment delivery and a reliable dose calculation algorithm to estimate the delivered dose. The log file‐based approach, indeed, offers many advantages over the conventional QA methods. However, regardless of what method we choose, the accuracy is of the utmost importance. There are still concerns and questions on the accuracy of the log file‐based QA approaches.

First, the accuracy of machine information recorded on the log file remains unclear. Furthermore, it is not clear to users whether the recorded information was measured with independent sensors; what is the accuracy and uncertainty of those sensors; whether we can perform adequate calibration and QA as we do for ion chambers and other QA devices; and whether there may exist failure modes for which the sensors fail to detect errors.

There are already a few incidents showing that the information recorded on log files failed to accurately reflect the actual machine behavior. One incident at a TomoTherapy site revealed that the jaw sizes were varying during rotational delivery while the jaw position recorded on the log file recorded the same position as planned. It turned out that the jaw was driven by a stepping motor and its connection was loose, leaving the jaw freely moving, whereas stepping motor positions recorded on the log file remained unchanged. After that event, TomoTherapy uses servo motor for the jaws, which contains position feedback, to prevent this incident from happening again. It has been speculated that the MLCs in Varian linacs may potentially have the same issue, since they use similar stepping motors for controlling MLCs. This was recently confirmed by Agnew et al.^(4)^ It was reported that the MLC position recorded on the log file does not agree with the observed MLC position due to a loose T‐nut.^(4)^ In both incidents, the errors were only caught by a measurement‐based QA method.

Secondly, the log file‐based method relies on a dose calculation algorithm in order to estimate the delivered dose. While well‐developed dose calculation algorithm, such as convolution–superposition, has been shown to produce fairly accurate results,^13^, ^14^, ^15^ there is always a degree of approximation in modeling the complex process of treatment delivery and dose deposition. Recently, it has been revealed that the discrete angular sampling (51 angles per rotation) used in TomoTherapy's dose engine is insufficient to model the continuous radiation of a small peripheral target, resulting in severe underdosing.^(16)^ The angular sampling rate used in tomotherapy dose calculation had been tripled to reduce this discretization error. Again, this error was caught by the measurement‐based method. If the dose calculation engine used by the log file‐based method contains the same approximation, this error is likely to go undetected.

Finally, there are several important aspects of treatment delivery that currently are not recorded in log files, such as profiles and energies of radiation beams. The change of those factors could have significant impact to the delivered dose, which can go undetected by the log file‐based approach. Note that, although the particular incidents mentioned in this article were either fixed or fixable by the manufacturer, they revealed possible loopholes in the log file‐based QA approach. Unless there are fundamental changes in this method, errors can go undetected by the log file‐based approach. The log file‐based QA approach offers many advantages, yet it still requires further investigation of its limitation before it is clinically adopted for IMRT QA.

### Dr. Nathan Childress

Dr. Chen argues against the use of log files for IMRT QA because data about the machine state contained in log files may be incorrect and a dose calculation algorithm must be used to derive information about the delivered dose.

With regard to the accuracy of log files, adherence to a rigorous linac QA protocol, such as AAPM TG‐142, ensures dose monitors, MLC position encoders, and other important delivery parameters are correctly calibrated. Since log files contain a complete record of the delivery parameters used by the linac to deliver patient treatments, their measurement data is correctly “calibrated”. If the physicist is not confident in the information recorded in log files reflecting the true behavior of the machine, that machine should not be eligible for patient treatments.

With regard to dose calculation algorithms, the accuracy of contemporary dose algorithms is thoroughly documented. For example, a well‐implemented collapsed cone algorithm has been shown to agree with measurements within 2%–5% in highly heterogeneous media.^17^, ^18^ If contemporary dose algorithms are not sufficiently accurate for IMRT QA purposes, they should not be used for treatment planning of actual patients.

The purpose of IMRT QA is to catch errors associated with the transfer and delivery of each patient's treatment plan, not to serve as part of the routine QA program. The conventional paradigm of IMRT QA requires an integrative test of the entire treatment chain (i.e., end‐to‐end test) each time. While this approach is sensitive to large machine errors, it is insensitive to the plan‐specific errors it is intended to detect, and it is a poor replacement for a routine QA program. In fact, the Imaging and Radiation Oncology Core at Houston (IROC Houston), formally the Radiological Physics Center (RPC), has found that conventional IMRT QA methods do not predict unacceptable plan deliveries.^(19)^

Log file‐based IMRT QA allows for analysis of dose in the patient CT using only the performance metrics of the linear accelerator. This enables physicists to isolate sources of error and directly determine the dosimetric impact of errors to the patient. When intelligently paired with a robust routine QA program, log file‐based IMRT QA significantly improves the efficiency, sensitivity, and specificity of patient‐specific QA.

### Dr. Quan Chen

I agree with Dr. Childress that log file‐based QA approach offers several superior features. However, it is still not certain whether the log file‐based QA approach offers the same confidence as measurements with ADCL‐calibrated ion chamber. Independent commissioning and robust routine QA may be required, as well as tests that can detect all possible failures. Nevertheless, the examples mentioned in my opening statement showed that there are vulnerability and weaknesses in hardware and software that went undetected for many years even though they went through extensive QA by manufacturer and clinical physicists. It is hard to establish a comprehensive QA method that can test all possible scenarios. Therefore, even with independent commissioning and routine QAs, dose predicted by log file‐based QA approach may still be less reliable than direct measurements.

I believe log file‐based method is still at its infant stage and cannot replace the conventional QA method. However, log file‐based QA can be a good complement to the conventional QA methods as it provides more details on treatment delivery. It is possible that as we accumulate more experience, we can identify more weaknesses and implement solutions to improve the reliability of the log file‐based QA. These improvements can be on the log file software itself, or on the commissioning and QA program, or on the treatment machine and treatment planning system by their manufacturers. After those necessary improvements and advances, we may have more confidence in answering the question whether the log file‐based QA can replace conventional QA methods for IMRT plans.
